# Association between PSCA gene polymorphisms and the risk of cancer: an updated meta-analysis and trial sequential analysis

**DOI:** 10.18632/oncotarget.17011

**Published:** 2017-04-10

**Authors:** Zhiqiang Qin, Jingyuan Tang, Xiao Li, Yajie Yu, Chuanjie Zhang, Peng Han, Ran Li, Xuping Jiang, Chengdi Yang, Wei Wang, Min Tang, Wei Zhang

**Affiliations:** ^1^ Department of Urology, The First Affiliated Hospital of Nanjing Medical University, Nanjing, 210029, China; ^2^ Department of Urology, Wuxi Second People's Hospital, Nanjing Medical University, Wuxi, 214002, China; ^3^ Department of Urologic Surgery, The Affiliated Cancer Hospital of Jiangsu Province of Nanjing Medical University, Nanjing, 210009, China; ^4^ First Clinical Medical College of Nanjing Medical University, Nanjing, 210029, China; ^5^ Department of Urology, Yixing People's Hospital, Wuxi, 214200, China

**Keywords:** PSCA polymorphisms, rs2294008, rs2976392, cancer, meta-analysis

## Abstract

Previous studies have investigated the relationships between PSCA rs2294008 C>T and rs2976392 G>A polymorphisms and cancer susceptibility. However, the available findings remained inconsistent and even controversial. Thus, the aim of this meta-analysis was performed to clarify such associations. The online databases PubMed, EMBASE and Web of Science searched for relevant studies, covering all the papers published until September 1st, 2016. Data were pooled by odds ratios (ORs) with 95% confidence intervals (CIs) to evaluate the strength of such associations. Then, trial sequential analysis was performed to estimate whether the evidence of the results was firm. Overall, a significant increased risk of cancer was associated with PSCA rs2294008 C>T and rs2976392 G>A polymorphisms. For the PSCA rs2294008 polymorphism, when stratified by type of cancer, the results were significant especially in gastric cancer and bladder cancer. Moreover, in the subgroup analysis by ethnicity, significant results were detected in both Asian and Caucasian populations. Similarly, for the PSCA rs2976392 polymorphism, the stratification analyses by type of cancer showed that the results were significant only in gastric cancer. In addition, the stratification analyses by ethnicity detected that this polymorphism increased cancer risk only in Asian populations. Then, trial sequential analyses demonstrated that the results of the meta-analysis were based on sufficient evidence. Therefore, this meta-analysis suggested that the PSCA rs2294008 C>T and rs2976392 G>A polymorphisms might be associated with cancer susceptibility, which might act as a potential predicted biomarker for genetic susceptibility to cancer, especially in gastric cancer and bladd er cancer.

## INTRODUCTION

Despite the obvious improvements in the early diagnosis and treatment of cancer, cancer remains amajor worldwide public health burden in recent year, with approximately 1,688,780 new cases and 600,920 new deaths in the United States in 2017 [1]. Cancer is a multi-step complex and multifactorial disease involving the intricate interactions between numerous genetic as well as environmental risk factors, such as age, race, lifestyle, obesity, family history, smoking status and endocrine system [2–4]. It is well known that various genes are associated with the carcinogenesis due to the polygenic inheritanc of cancer [5]. However, the exact mechanism of cancer is unclear and remains to be identified. Multiple studies have shown that the screening and identification of single-nucleotide polymorphisms (SNPs) as a predicted biomarker of human genetic variation might affect individual in the sensitivity to cancer risk and therapeutic responses in early cancer patients. Therefore, it has been demonstrated that SNPs may play an important role in high susceptibility for cancer to discover novel loci or genes [6].

As a LY-6/Thy-1 family of cell surface antigens, prostate stem cell antigen (PSCA) gene is located on chromosome 8q24.2 containing 464 SNPs, and the PSCA protein is a 123-amino-acid cell membrane glycoprotein, which encodes a PSCA protein that is reported as a cell surface marker [7]. Compared to the normal tissues, PSCA is further up-regulated in prostate cancer tissue, and which is also found in several other cancers, including pancreatic cancer and gallbladder cancer [8–10]. Moreover, as the most extensively studied SNPs in the PSCA gene, rs2294008 C>T and rs2976392 G>A are shown to be associated with increased risk of bladder and stomach cancers [11, 12]. However, there is no obvious evidence for the role of PSCA in carcinogenesis. Thus, it was hypothesized that PSCA gene polymorphisms were likely to play an vital role in carcinogenesis.

In recent years, several studies have been widely investigated the possible association between the PSCA polymorphisms and risk of cancer. For instance, Qiu et al. [13] demonstrated that the PSCA rs2294008 T alleles was risk factors for gastric cancer in this eastern Chinese population. However, Mou et al. [19] found that PSCA rs2294008 polymorphism possessed no difference/association with gastric cancer risk among cases and controls. Hence, we cannot definitively declare that the observed association between PSCA polymorphisms and risk of cancer. So, we aimed to conduct a meta-analysis including all accessible case-control studies to reconcile all the discordant results to systematically clarify the role of these SNPs in susceptibility to cancer. Additionally, lack of further research in trial sequential analysis (TSA) prevented comprehensive understanding of the association between PSCA polymorphisms and cancer susceptibility in some previous meta-analyses. Hence, with this in mind, we conducted the present meta-analysis and TSA to critically evaluate the association between PSCA rs2294008 C>T and rs2976392 G>A polymorphisms and cancer risk and clarify whether the evidence for the results was sufficient.

## RESULTS

### Studies characteristics

**Figure 1:**
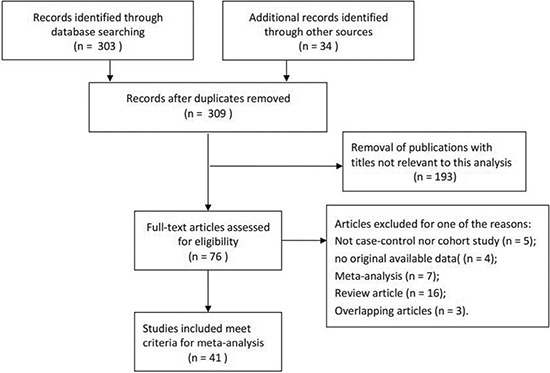
Flowchart of literature search and selection process

A total of 41 case-control studies were found to fulfill the eligibility criteria for the current meta-analysis of the PSCA rs2294008 and rs2976392 with cancer risk including 34,764 patients and 43,309 controls [11–48], and the detailed characteristics of individual studies included were listed in Table 1. Besides, the distribution of genotypes in the controls was consistent between Hardy-Weinberg equilibrium (HWE) in all involved studies except two articles [13, 37]. Figure 1 showed the flowchart of literature search and selection process.

**Table 1:** Characteristics of individual studies included in the meta-analysis

PSCA	rs2294008								Case(n)			Control(n)				
Year	Author	Country	Ethnicity	SOC	Genotyping	Type of Cancer	Case	Control	CC	CT	TT	CC	CT	TT	NOS points	HWE
2016	Qiu	China	Asian	HB	Taqman	Gastric	1124	1192	537	489	98	663	383	146	8	N
2016	Wang	China	Asian	HB	Sequenom	Breast	560	583	273	231	56	299	247	37	8	Y
2016	Wang	China	Asian	HB	Taqman	Cervical	1126	1237	609	469	48	618	527	92	9	Y
2015	Garcia-Gonzalez	Spain	Caucasian	HB	Taqman	Gastric	603	675	154	302	147	199	346	130	9	Y
2015	Ichikawa	Japan	Asian	HB	PCR-RFLP	Gastric	193	266	24	104	65	52	119	95	7	Y
2015	Sun	China	Asian	HB	Taqman	Gastric	692	774	322	309	61	405	297	72	9	Y
2015	Kupcinskas	Latvia	Caucasian	HB	Taqman	Colorectal	191	377	60	77	54	100	189	88	7	Y
2015	Zhang	China	Asian	HB	Sequenom	Gastric	475	480	227	207	41	261	183	36	8	Y
2015	Mou	China	Asian	PB	DHPLC	Gastric	198	130	23	126	49	5	34	91	9	Y
2014	Kupcinskas	Lithuania	Caucasian	HB	Taqman	Gastric	251	243	33	116	102	64	123	56	8	Y
2014	Lee	Korea	Asian	HB	HRM	Bladder	411	1700	70	222	119	414	818	468	9	Y
2014	Wang	China	Asian	PB	Taqman	Bladder	1210	1008	604	509	97	566	376	66	9	Y
2014	Sun	USA	Caucasian	HB	Taqman	Gastric	130	125	17	64	49	30	63	32	9	Y
2014	Dai	China	Asian	PB	Taqman	Esophageal	2083	2220	1232	724	127	1222	851	147	9	Y
2013	Zhao	China	Asian	PB	DHPLC	Gastric	717	951	275	342	100	465	401	85	8	Y
2013	Rizzato	Germany	Caucasian	PB	Taqman	Gastric	178	1057	23	86	69	231	507	319	9	Y
2013	Rai	India	Asian	HB	Taqman	Gallbladder	405	247	104	233	68	79	126	42	7	Y
2013	Ono	Japan	Asian	HB	Taqman	Gallbladder	44	173	9	23	12	30	75	68	8	Y
2013	Ma	China	Asian	PB	MassARRAY	Bladder	175	962	84	80	11	543	355	64	9	Y
2012	Smith	Scotland	Caucasian	HB	Taqman	Colorectal	77	804	25	39	13	287	387	130	7	Y
2012	Sala	European	Caucasian	PB	Taqman	Gastric	409	1515	93	198	118	491	714	310	9	Y
2012	Li	China	Asian	PB	MassARRAY	Gastric	300	300	124	141	35	168	111	21	8	Y
2012	Kim	Korea	Asian	HB	MassARRAY	Breast	451	459	119	216	116	113	240	106	8	Y
2012	Fu	European &USA	Caucasian	PB	GWAS	Bladder	5393	7324	1363	2804	1226	2107	3645	1572	9	Y
2011	Zeng	China	Asian	HB	PCR-RFLP	Gastric	460	549	202	216	42	289	223	37	8	Y
2011	Song	Korea	Asian	HB	PCR-RFLP	Gastric	3245	1700	576	1620	1049	414	818	468	9	Y
2011	Lochhead	USA	Caucasian	PB	Taqman	Esophageal	158	208	61	63	34	49	110	49	9	Y
2011	Lochhead	USA	Caucasian	PB	Taqman	Gastric	308	208	85	129	94	49	110	49	9	Y
2011	Lochhead	Poland	Caucasian	PB	Taqman	Gastric	292	382	47	143	102	101	166	115	8	N
2011	Joung	Korea	Asian	HB	MassARRAY	Prostate	192	168	45	98	49	47	84	37	9	Y
2010	Wang	China	Asian	HB	PCR-RFLP	Bladder	581	580	272	259	50	316	220	44	7	Y
2010	Ou	China	Asian	HB	PCR/LDR	Gastric	196	246	85	93	18	132	96	18	8	Y
2010	Lu	China	Asian	PB	PCR-RFLP	Gastric	1023	1069	547	404	72	605	387	77	8	Y
2009	Wu	European&USA	Caucasian	HB	GWAS	Bladder	5038	9363	1288	2613	1137	2842	4668	1853	9	Y
2009	Wu	China	Asian	PB	PCR-RFLP	Gastric	1710	995	759	819	132	506	412	77	9	Y
2009	Matsuo	Japan	Asian	HB	Taqman	Gastric	708	708	330	329	49	273	338	97	8	Y
2008	Sakamoto	Korea	Asian	HB	Taqman	Gastric	871	390	133	461	277	122	176	92	7	Y
2008	Sakamoto	Japan	Asian	HB	GWAS	Gastric	1524	1396	96	700	728	210	650	536	9	Y
**PSCA**	**rs2976392**															
Year	Surname	Country	Ethnicity	SOC	Genotyping	Type of Cancer	Case	Control	GG	GA	AA	GG	GA	AA		HWE
2016	Qiu	China	Asian	HB	Taqman	Gastric	1124	1192	535	488	101	682	388	122	8	N
2016	Wang	China	Asian	HB	Sequenom	Breast	560	583	287	230	43	298	247	38	8	Y
2015	Kupcinskas	Latvia	Caucasian	HB	Taqman	Colorectal	191	364	56	84	51	99	180	85	7	Y
2015	Sun	China	Asian	HB	Taqman	Gastric	692	774	319	308	65	403	299	72	9	Y
2015	Zhang	China	Asian	HB	Sequenom	Gastric	436	451	190	208	38	231	184	36	8	Y
2014	Kupcinskas	Lithuania	Caucasian	HB	Taqman	Gastric	249	232	34	113	102	62	116	54	8	Y
2014	Wang	China	Asian	HB	Taqman	Gastric	283	275	131	134	18	149	108	18	9	Y
2013	Ju	China	Asian	HB	sequencing	Gastric	155	210	67	65	23	107	87	16	8	Y
2013	Ono	Japan	Asian	HB	Taqman	Gallbladder	44	173	9	23	12	29	76	68	8	Y
2012	Kim	Korea	Asian	HB	MassARRAY	Breast	453	460	121	217	115	115	239	106	8	Y
2011	Shen	China	Asian	PB	DHPLC	Gastric	60	60	24	31	5	29	26	5	9	Y
2011	Joung	Korea	Asian	HB	MassARRAY	Prostate	194	168	45	100	49	46	85	37	9	Y
2010	Ou	China	Asian	HB	PCR/LDR	Gastric	196	246	99	85	12	130	102	14	8	Y
2010	Lu	China	Asian	PB	PCR-RFLP	Gastric	1043	1082	500	464	79	602	402	78	8	Y
2009	Wu	China	Asian	PB	PCR-RFLP	Gastric	1724	1002	789	793	142	492	429	81	9	Y
2009	Matsuo	Japan	Asian	HB	Taqman	Gastric	707	707	331	328	48	274	337	96	8	Y
2008	Sakamoto	Korea	Asian	HB	Taqman	Gastric	865	390	134	453	278	122	175	93	7	Y
2008	Sakamoto	Japan	Asian	HB	GWAS	Gastric	1525	1397	97	691	737	211	650	536	9	Y

SOC: source of control; HB: hospital-based controls; PB: population-based controls;

NOS: Newcastle-Ottawa Scale; HWE: Hardy-Weinberg equilibrium.

For the PSCA rs2294008 polymorphism, 38 studies were performed on investigating the association between this SNP with susceptibility of cancer, including 34,266 cases and 42,764 controls [11–45]. In these studies, there were 26 studies of Asian populations and the other 12 studies were Caucasian ethnicity. The studied type of cancer included gastric cancer, breast cancer, cervical cancer, colorectal cancer, bladder cancer, esophageal cancer, gallbladder cancer and prostate cancer. Besides, seven genotyping methods were applied, such as Taqman, Sequenom, PCR-RFLP, DHPLC, HRM, GWAS and PCR/LDR. Furthermore, we divided them into population-based group or hospital-based group in all studies to distinguish between different sources of control group. Similarly, for the PSCA rs2976392 polymorphism, there were 18 studies exploring the relationship between this polymorphism and risk of overall cancer with 10,501 cases and 9,766 controls [12–14, 18, 20–21, 23, 28, 32, 36, 40–41, 43–44, 46–48]. In regard to source of control, the studies consisted of 3 population-based controls and 15 hospital-based controls. Moreover, there were 16 Asian populations and 2 Caucasian populations. In addition, the studied cancer type included gastric cancer, prostate cancer, colorectal cancer, breast cancer, and gallbladder cancer.

### Quantitative synthesis results

**Figure 2:**
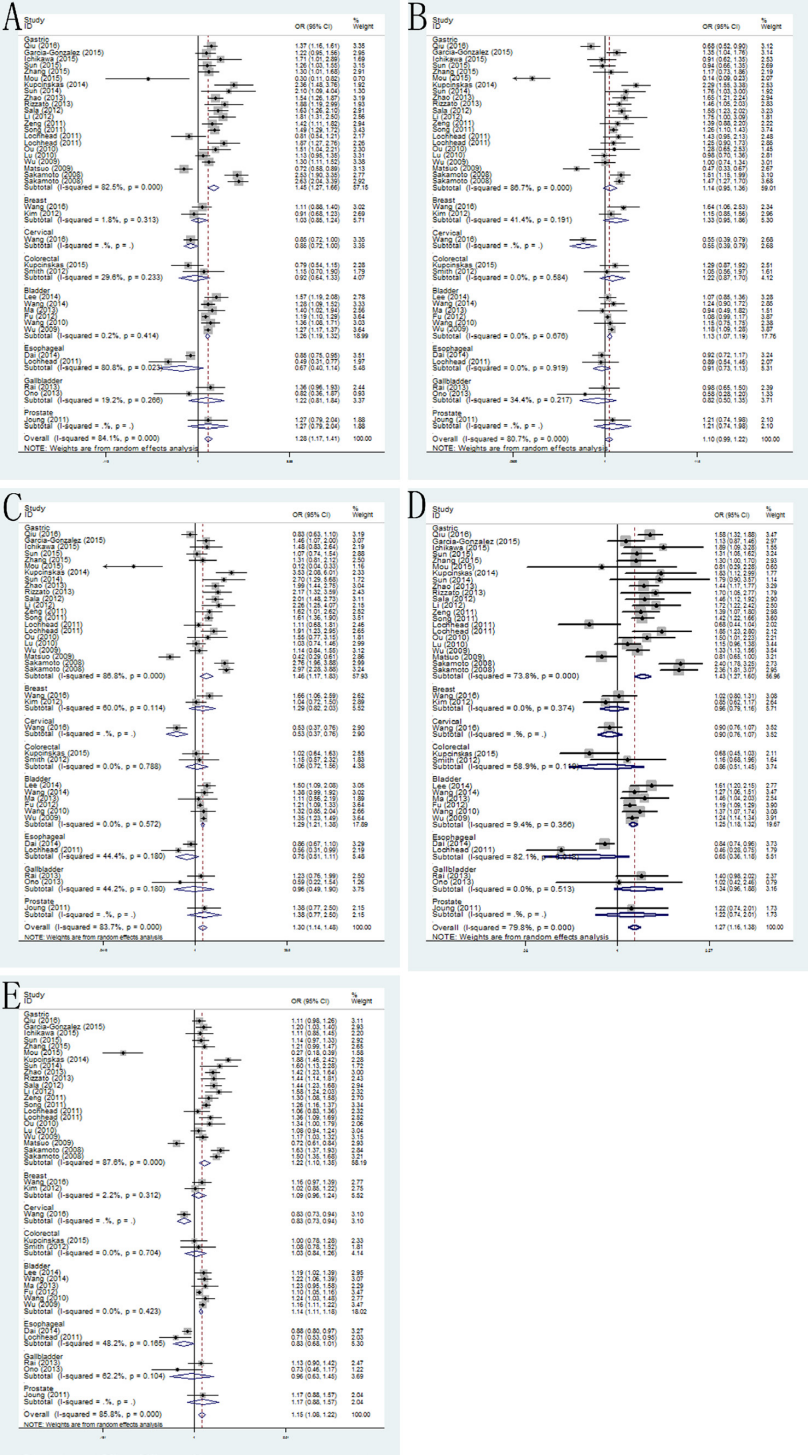
Forest plots of the association between PSCA rs2294008 C>T polymorphism and cancer susceptibility in the stratification analyses by type of cancer (**A**) dominant model; (**B**) recessive model; (**C**) homozygous model; (**D**) heterozygous model; (**E**) allele model.

The main results of this meta-analysis of the associations between PSCA rs2294008 C>T and rs2976392 G>A polymorphisms and risk of cancer were showed in Supplementary Table 1. Overall, our results indicated that PSCA rs2294008 C>T polymorphism was associated with an increased risk of cancer (*dominant model*: pooled OR=1.28, 95% CI: 1.17–1.41; *recessive model*: pooled OR=1.10, 95% CI: 0.99–1.22; *homozygote model*: pooled OR=1.30 95% CI: 1.14–1.48; *heterozygote model*: pooled OR=1.27, 95% CI: 1.14–1.48; *allele model*: pooled OR=1.15, 95% CI: 1.08–1.22) in the random-effects model. When stratified by type of cancer, the results showed PSCA rs2294008 had significantly increased risk of gastric cancer (*dominant model*: pooled OR = 1.45, 95% CI: 1.27–1.66; *recessive model*: pooled OR = 1.14, 95% CI: 0.95–1.36; *homozygote model*: pooled OR = 1.46 95% CI: 1.17–1.83; *heterozygote model*: pooled OR = 1.43, 95% CI: 1.27–1.60; *allele model*: pooled OR = 1.22, 95% CI: 1.10–1.35) and bladder cancer (*dominant model*: pooled OR = 1.26, 95% CI: 1.19–1.32; *recessive model*: pooled OR = 1.18, 95% CI: 1.07–1.19; *homozygote model*: pooled OR=1.29 95% CI: 1.21–1.38; *heterozygote model*: pooled OR=1.25, 95% CI: 1.18–1.32; *allele model*: pooled OR=1.14, 95% CI: 1.07–1.19) (Figure 2). Besides, the stratification analyses by ethnicity found that the results were significant in Asian and Caucasian populations. What's more, in the subgroup analysis by source of controls, carriers of T allele in PSCA rs2294008 were a strong risk factor of cancer in both population-based controls and hospital-based controls.

**Figure 3:**
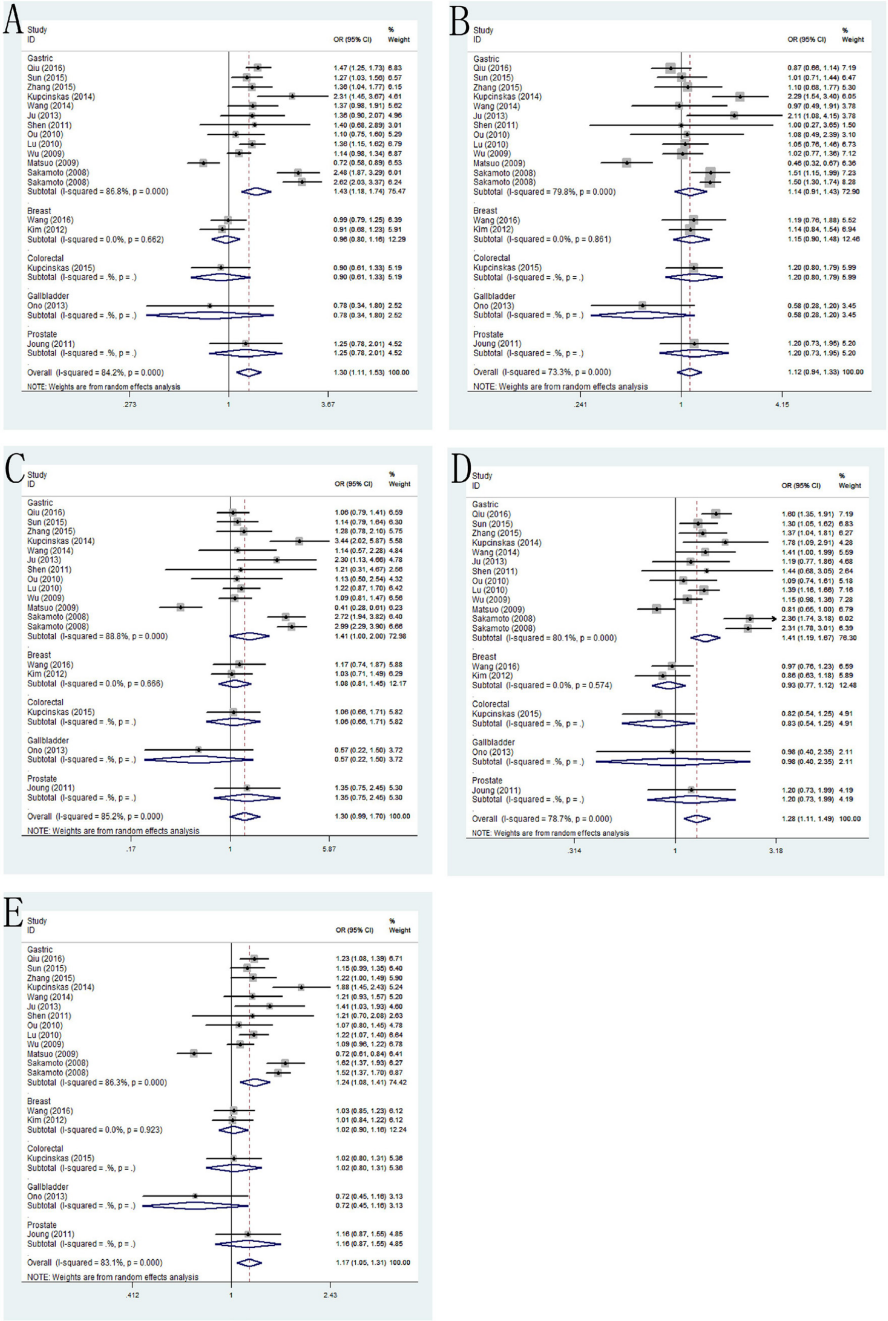
Forest plots of the association between PSCA rs2976392 G>A polymorphism and cancer susceptibility in the stratification analyses by type of cancer (**A**) dominant model; (**B**) recessive model; (**C**) homozygous model; (**D**) heterozygous model; (**E**) allele model.

In the PSCA rs2976392 polymorphism, we found this polymorphism was significantly associated with risk of cancer (*dominant model*: pooled OR=1.30, 95% CI: 1.11–1.53; *recessive model*: pooled OR=1.12, 95% CI: 0.94–1.33; *homozygote model*: pooled OR=1.30 95% CI: 0.99–1.70; *heterozygote model*: pooled OR=1.28, 95% CI: 1.11–1.49; *allele model*: pooled OR=1.17, 95% CI: 1.05–1.31). Stratification analyses by type of cancer also detected that rs2976392 polymorphism increased cancer risk only in gastric cancer (*dominant model*: pooled OR=1.43, 95% CI: 1.18–1.74; *recessive model*: pooled OR=1.14, 95% CI: 0.91–1.43; *homozygote model*: pooled OR=1.41 95% CI: 1.23–1.61; *heterozygote model*: pooled OR=1.41, 95% CI: 1.19–1.67; *allele model*: pooled OR=1.24, 95% CI: 1.08–1.41) (Figure 3). Moreover, in the stratification analyses by ethnicity, the significant results were only in Asian populations (*dominant model*: pooled OR=1.29, 95% CI: 1.09–1.53; *recessive model*: pooled OR=1.06, 95% CI: 0.89–1.27; *homozygote model*: pooled OR=1.24 95% CI: 0.93–1.64; *heterozygote model*: pooled OR=1.29, 95% CI: 1.11–1.51; *allele model*: pooled OR=1.15, 95% CI: 1.03–1.29). Lastly, increased cancer susceptibility associated with PSCA rs2976392 was also observed in population-based and hospital-based studies.

### Sensitivity analysis

Sensitivity analysis was carried out to distinguish their influence of each individual study on the combined values by repeating the meta-analysis through sequentially deleting the single studies study each time. The sensitivity analysis of associations for PSCA rs2294008 C>T and rs2976392 G>A polymorphisms with the risk of cancer in five types of models (dominant model, recessive model, homozygous model, heterozygous model and allele model) was listed in Supplementary Figure 1, which demonstrated stability and reliability of results for such associations.

### Publication bias

**Figure 4:**
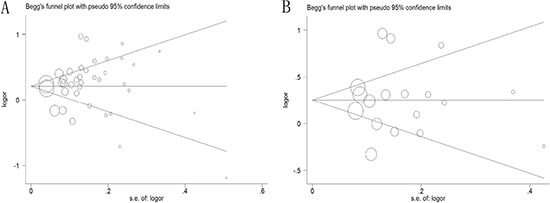
Begg's funnel plot of publication bias test in dominant model (**A**) PSCA rs2294008 C>T polymorphism; (**B**) PSCA rs2976392 G>A polymorphism.

We assessed the potential publication bias for the all available data by the Begg's funnel plot and Egger's test and the results were shown in Figure 4. No symmetric distribution was seemed in the shapes of the funnel plots for the dominant model, indicating no evidence of significant publication bias, which was also confirmed using Egger's test (rs2294008 C>T: *P* = 0.423; rs2976392 G>A: *P* = 0.842).

### Trial sequential analysis results

**Figure 5:**
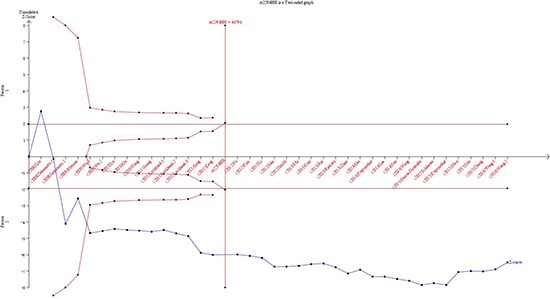
Trial sequential analysis of the association between PSCA polymorphisms and the risk of cancer The required information size was calculated based on a two side α = 5%, β = 20%, and a 95% confidence intervals. (**A**) PSCA rs2294008 C>T polymorphism; (**B**) PSCA rs2976392 G>A polymorphism.

Subsequently, the cumulative Z-curve exceeded the monitoring boundaries and the information size in the PSCA rs2294008 polymorphism by TSA, suggesting sufficient evidence of such association. In addition, the results in the PSCA rs2976392 polymorphism were proved to be solid with sufficient evidence, because of exceeding the trial sequential monitoring boundary. As a result, this findings revealed PSCA rs2294008 C>T and rs2976392 G>A polymorphisms were strongly associated with cancer risk (Figure 5).

## DISCUSSION

The PSCA gene belongs to a member of Ly-6/Thy-1 family of glycosylphosphatidyl-inositol (GPI)-anchored cell-surface proteins and plays a critical role in multiple cellular events, including cell adhesion, proliferation, and survival [7]. The over-expression of PSCA was initially reported in prostate cancer [36]. Besides, its high-expression is significantly associated with poor prognosis including seminal vesicle invasion, capsular involvement and Gleason score [49]. Therefore, PSCA has been considered as a biomarker of diagnosis and prognosis, as well as a target of therapy for prostate cancer. Moreover, some solid cancer including ovarian mucinous tumor, pancreatic cancer, renal cell carcinoma and bladder cancer have also existed the expression of PSCA [50]. In contrast with observations in prostate cancer, PSCA expression is down-regulated in several cancers, such as gastric cancer, bladder cancer, and gallbladder carcinoma [13]. Morover, PSCA may have tumor-suppressing function in the gastric epithelium in these specific type of cancers.

Previous studies have investigated the associations of PSCA polymorphisms with various cancer susceptibility [51–53]. For instance, Chandra et al. [52] demonstrated that the PSCA polymorphisms was risk factors for cancer in Asian Population. Besides, Gao et al. [53] found that PSCA rs2294008 polymorphism possessed association with bladder cancer risk. Nevertheless, these results are discrepant and even conflicting. A possible reason arised from the differences in study design, sample size, source of controls, race and genotyping method. All these contributed to the limited statistical power in the published studies. Hence, as we included more studies about the associations between PSCA polymorphisms and the risk of cancer, this meta-analysis was carried out to provide more reliable conclusion to reveal the real associations compared the previous meta-analyses [51]. Furthermore, TSA was used to clarify whether the evidence for the results was sufficient.

As a powerful tool, meta-analysis can provide more sufficient results compared to a single study especially in analyzing unexplained studies [54]. As a result, we suggested there existed a much stronger advantage to prove the association between PSCA rs2294008 C>T and rs2976392 G>A polymorphisms with the susceptibility to cancer. On the one hand, further researches in different stratified analysis were necessary in these meta-analyses. On the other hand, we for the first time applied TSA to reduce the risk of type I error and testify whether the evidence of our results was reliable. The results suggested that significantly elevated cancer risk was associated with the PSCA rs2294008 C>T polymorphism levels, particularly in patients with gastric cancer and bladder cancer. Meanwhile, PSCA rs2976392 G>A polymorphism significantly increased cancer risk only in gastric cancer.

After stratified analysis by type of cancer, the results showed PSCA rs2294008 C>T and rs2976392 G>A polymorphisms statistically increased cancer risk, especially in gastric cancer and bladder cancer instead of breast cancer, cervical cancer, colorectal cancer and other cancers. Different kinds of cancer have specific characteristic of diverse aspects, which might lead to different statistical results. In addition, the different type of cancer has distinctive polymorphism sites. Therefore, only specific polymorphism site might be associated with a certain type of tumor.

These findings of subgroup analyses based on ethnicity and source of control can be explained as follows. In the subgroup analysis by ethnicity, significantly increased cancer risk was shown in Asian and Caucasian populations in PSCA rs2294008 C>T polymorphism. Besides, PSCA rs2976392 G>A polymorphism increased risk of cancer only in Asian populations. Though the exact mechanism was unclear, it was possible that different ethnic groups with various genetic backgrounds might have different SNPs in the developing of cancer. Meanwhile, it is important to meet the unified enrollment criteria and select larger sample size studies, which could make the results more reliable. In addition, we conducted stratified analysis by source of controls and the result was detected significantly both in population-based and hospital-based populations. In this meta-analysis, the results were in concordance with these hypotheses of previous studies, which needed to further prove that PSCA rs2294008 and rs2976392 played an important role in cancer susceptibility as far as possible in all relevant articles published in the future.

As an useful approach, TSA is introduced to calculate the required information size for this meta-analysis with the adaptation of monitoring boundaries, in order to reduce the risk of type I error [55–57]. Besides, we took advantage of TSA with all included trials to estimate whether a sufficient level of evidence had been reached and whether further trials were necessary [58–60]. When a *P* value is sufficiently small to show the anticipated effect, it is believed that the application of TSA shows the potential to be more reliable compared to the traditional meta-analysis. For findings of risk of cancer in PSCA rs2294008 C>T polymorphism, the cumulative Z-curve crossed not only the monitoring boundaries but also the sufficient information size, suggesting that additional new clinical trial should not be needed. Moreover, for results of risk of cancer in PSCA rs2976392 G>A polymorphism, the cumulative Z-curve not exceeding the required sample size crossed the trial sequential monitoring boundaries, which indicated that our conclusion had reached a sufficient level of evidence [61,62]. In consequence, it was strongly of the view that our results in the current meta-analysis were based on firm evidence of effect, and no further studies was needed to investigate such associations.

Although the overall robust statistical evidence including the implementation of TSA was to estimate a slight association by this meta-analysis, some limitations of this meta-analysis should be taken into consideration when interpreting the present results. Firstly, some published studies involved in the PSCA polymorphisms are not accord with the HWE, resulting in potential bias during control selection or genotyping errors. Secondly, because of existing significant heterogeneity in this meta-analysis, it was very likely the results were interpreted. Thirdly, the effect of multiple confounders such as age, gender, life-style may also play an important role in the development of cancer, but we could not make these subgroup analysis due to insufficient data on the basis of these factors. What's more, as a multi-factorial disease, the pathogenesis of cancer is closely related complex interactions between a variety of genetic factors and environmental backgrounds, suggesting risk of cancer would not be influenced by any single gene. Therefore, more new-designed studies about exploring the risk effects of these two SNPs in susceptibility to cancer needed to be further validated in subsequent studies. Accordingly, it is required that more studies be conducted to provide a more definitive conclusion.

## MATERIALS AND METHODS

A comprehensive literature search was systematically conducted using the electronic databases PubMed, EMBASE and Web of Science for potential relevant studies, which investigated the association between PSCA rs2294008 C>T and rs2976392 G>A polymorphisms and risk of cancer, covering all the papers published until September 1st, 2016. The combinations of the following keywords were used: “prostate stem cell antigen”, “PSCA polymorphisms”, “rs2294008” or “rs2976392”, and “gene”, “variant”, “polymorphism” or “mutation”, and “caner”, “carcinoma” or “neoplasms”. Eligible literatures were retrieved from all publications. Besides, additional literature was further collected manually from reference lists of reviews to make sure all potential eligible publications. Moreover, if studies had partly familiar or overlapping subjects, only the latest or largest sample size was adopted in this meta-analysis.

### Inclusion and exclusion criteria

Studies were included if they met the inclusion criteria as follows: (1) a case-control or cohort design; (2) investigate or report the relationship between PSCA polymorphisms and cancer susceptibility; (3) sufficient genotype frequency data provided to calculate the odds ratio (OR) and 95% confidence interval (CI). In addition, the major excluding criterion was as follows: (1) no relevant genotype frequency data or overlapping data; (2) reviews or conference abstracts; (3) no case-control studies; (4) providing duplicates of previous publication with others.

### Data extraction

According to the eligibility criteria, data were extracted from each manuscript independently by two investigators (Qin ZQ and Tang JY). Besides, any disagreement would be solved by a discussion with a third investigator (Li X) to reach a consensus on all the extracted information. From each article, first author's name, year of publication, country, ethnicity, source of controls (population-based or hospital-based), genotyping method, type of cancer, sample size of cases and controls, frequency of PSCA rs2294008 and rs2976392 gene polymorphisms in cases and controls respectively, and the results of the HWE test were recorded in a standardized form.

### Quality assessment

The quality of the studies was assessed using the validated Newcastle-Ottawa Scale (NOS) for nonrandomized studies, including case-control and cohort studies. Separate NOS scales were developed for cohort and case-control studies. It has not been published in peer-reviewed journals so far, although NOS has been widely utilized. NOS awards eight points to each case-control study (four for quality of selection, one for comparability, and three for quality of exposure). A study can be awarded a maximum of one star for each point within the selection and exposure categories, and a maximum of two stars can be given for comparability. Besides, NOS also awards eight points to each cohort study (four for quality of selection, one for comparability, and three for quality of outcome). A study can be awarded a maximum of one star for each point within the selection and outcome categories, and a maximum of two stars can be given for comparability. We considered studies with scores of more than 7 as high-quality studies, and those with scores of 7 or less as low-quality studies.

### Statistical analysis

The crude odds ratios (ORs) with 95% confidence intervals (CIs) were measured to evaluate the strength of association between the PSCA rs2294008 and rs2976392 gene polymorphisms with overall cancer susceptibility under these five genetic comparison models: dominant model, recessive model, homozygous model, heterozygous model and allele model, based on the genotype frequency distribution in cases and controls. An OR value > 1 indicated a significantly increased cancer risk, while an OR value < 1 stood for more benefit in risk of cancer. The goodness-of-fit chi-square test was adopted to check HWE among controls, and the deviation was regarded significant disequilibrium at the 0.05 level. The between-study heterogeneity was estimated using the chi-square-based Q test and quantified with the I^2^ statistic. When *P* < 0.05 was considered the presence of significant heterogeneity among studies, the random-effects model (DerSimonian-Laird method) would be conducted. In addition, the pooled OR was calculated using the fixed-effects model (Mantel-Haenszel method) in the absence of heterogeneity. After that, subgroup analysis was further performed by type of cancer, ethnicity and source of controls. In sensitivity analysis, each study was omitted each time and the pooled ORs with 95% CIs were recalculated to measure the stability of pooled results. Publication bias between the studies was performed using Begg's funnel plots and Egger's linear regression test and by visual inspection of the funnel plot. All *P* values were two-sided and a *P* < 0.05 was considered statistically significant. All statistical data was carried out by Stata software (version 12.0; StataCorp LP, College Station, TX).

### Trial sequential analysis

Outcome of meta-analysis might be prone to systematic or random errors owing to repeated significance testing of accumulated data and collecting sparse data, when cumulative meta-analyses were updated with addition of new publishing studies [56, 60, 63]. Thus, TSA was performed to reduce the risk of type I errors and confirmed more statistical reliability of the data by estimation of required information size with an adjusted threshold for statistical significance [57, 58]. In the current meta-analysis, TSA was performed with a desire to maintain a 95% confidence intervals, a 20% relative risk reduction, an overall 5% risk a type I error and a statistical test power of 80% (20% risk of the type II error), which meant that the required information size was calculated and the trial sequential monitoring boundaries was constructed. When the cumulative Z-curve (the blue line) crossed the trial sequential monitoring boundary (sloping red line) or exceeded the required information size (vertical red line), a sufficient level of evidence might have been reached and no further studies were needed. Otherwise, if the blue line did not cross any of the boundaries and the vertical red line has not been reached, additional clinical trials are needed to obtain sufficient evidence by reaching the adequate required information size [59–60, 64]. The trial sequential analysis software (TSA, version 0.9; Copenhagen Trial Unit, Copenhagen, Denmark, 2011) was carried out in this study.

## CONCLUSIONS

This current meta-analysis provided statistical evidence supporting that the PSCA rs2294008 C>T and rs2976392 G>A polymorphisms increased the risk of cancer, especially in gastric cancer and bladder cancer. Therefore, the PSCA s2294008 C>T and rs2976392 G>A polymorphisms might be considered an ideal marker in the prediction of cancer in the subsequent studies. Nevertheless, more well-designed studies need to be further checked with a sufficiently large number of participants to substantiate these real associations.

### Ethical statements

None declared.

## SUPPLEMENTARY MATERIALS FIGURE AND TABLE




