# Supplementary material for: Association between PSCA gene polymorphisms and the risk of cancer: an updated meta-analysis and trial sequential analysis
Source: Oncotarget. 2017 Apr 10;8(31):51766–78. doi: 10.18632/oncotarget.17011 (PMC5584286; doi:10.18632/oncotarget.17011)
Supplement: Supplementary file 1 [file oncotarget-08-51766-s001.pdf]

Association between PSCA gene polymorphisms and the risk of cancer: an updated meta-analysis and trial sequential analysis

Supplementary Materials

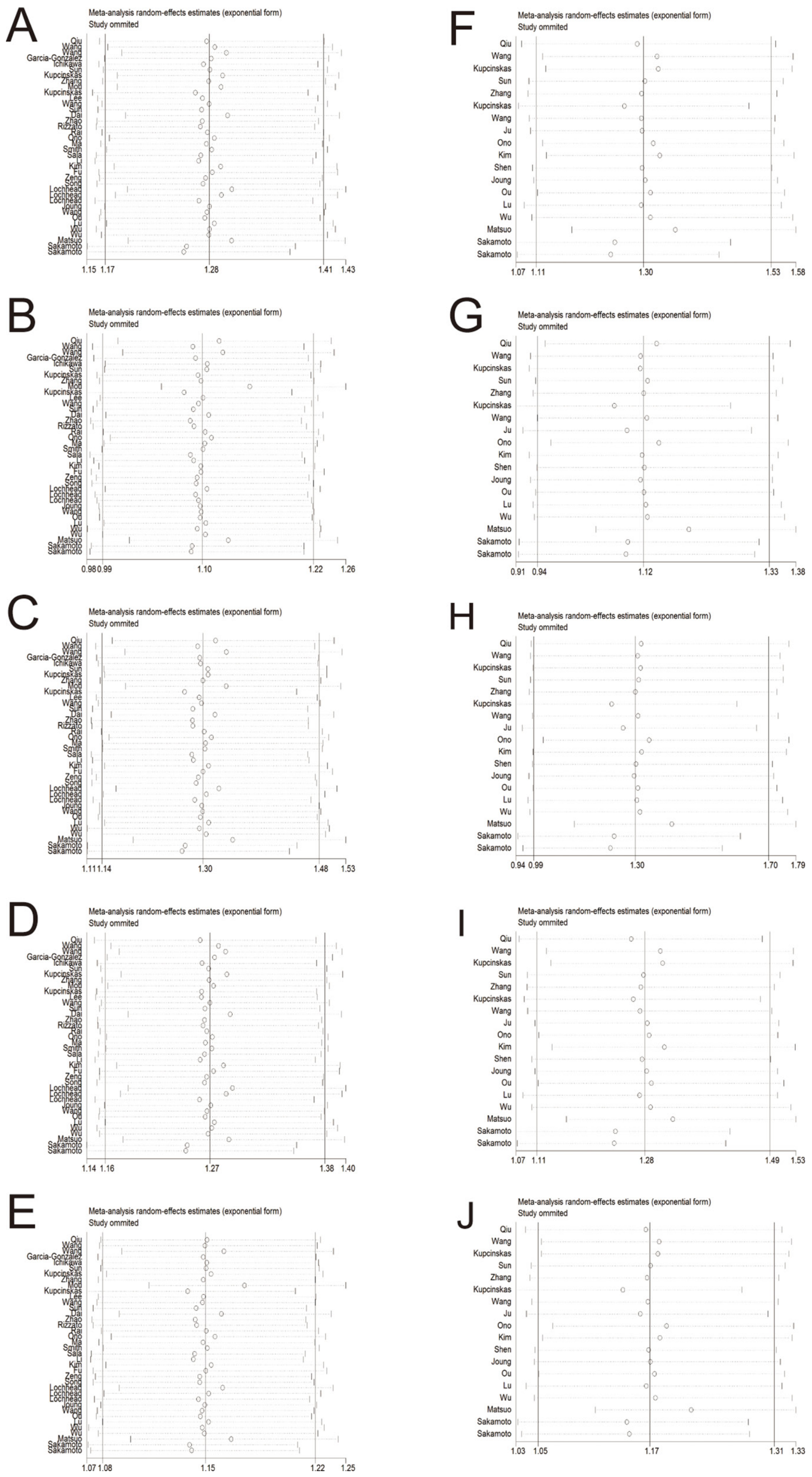

Supplementary Figure 1: Sensitivity analysis under five types of models (dominant model, recessive model, homozygous model, heterozygous model and allele model). (**A–E**) PSCA rs2294008 C>T polymorphism under dominant model, recessive model, homozygous model, heterozygous model and allele model, respectively; (**F–J**) PSCA rs2976392 G>A polymorphism under dominant model, recessive model, homozygous model, heterozygous model and allele model, respectively.

Supplementary Table 1: Meta-analysis results for the included studies of the association between PSCA rs2294008 and rs2976392 polymorphisms and risk of cancer. See Supplementary_Table_1
