# Supplementary material for: Association between PSCA gene polymorphisms and the risk of cancer: an updated meta-analysis and trial sequential analysis
Source: Oncotarget. 2017 Apr 10;8(31):51766–78. doi: 10.18632/oncotarget.17011 (PMC5584286; doi:10.18632/oncotarget.17011)
Supplement: Supplementary file 2 [file oncotarget-08-51766-s002.doc]

| Table 2: Meta-analysis results for the included studies of the association between PSCA rs2294008 and rs2976392 polymorphisms and risk of cancer | | | | | | | | | | | | | | | | | |
| --- | --- | --- | --- | --- | --- | --- | --- | --- | --- | --- | --- | --- | --- | --- | --- | --- | --- |
| Variables | No. of studies | Dominant model | | | Recessive model | | | Homozygous model | | | Heterozygous model | | | Allele model | | |  |
|  |  | OR (95% CI) | P values | I-squared (%) | OR (95% CI) | P values | I-squared (%) | OR (95% CI) | P values | I-squared (%) | OR (95% CI) | P values | I-squared (%) | OR (95% CI) | P values | I-squared (%) |  |
| ***rs2294008 C>T*** |  | (CT + TT) vs. CC | | | TT vs. (CT + CC) | | | TT vs. CC | | | CT vs. CC | | | T vs. C | | |  |
| All | 38 | 1.28 (1.17-1.41) | <0.001 | 84.1 | 1.10 (0.99-1.22) | <0.001 | 80.7 | 1.30 (1.14-1.48) | <0.001 | 83.7 | 1.27 (1.16-1.38) | <0.001 | 79.8 | 1.15 (1.08-1.22) | <0.001 | 85.8 |  |
| Ethnicity |  |  |  |  |  |  |  |  |  |  |  |  |  |  |  |  |  |
| Asian | 26 | 1.29 (1.14-1.46) | <0.001 | 86.4 | 0.99 (0.85-1.17) | <0.001 | 84.4 | 1.20 (0.99-1.47) | <0.001 | 86.3 | 1.31 (1.17-1.47) | <0.001 | 82.2 | 1.11 (1.01-1.22) | <0.001 | 88.3 |  |
| Caucasian | 12 | 1.26 (1.08-1.46) | <0.001 | 77.5 | 1.31 (1.17-1.47) | 0.003 | 60.7 | 1.48 (1.24-1.76) | <0.001 | 75.2 | 1.16 (1.01-1.35) | <0.001 | 73.8 | 1.21 (1.11-1.33) | <0.001 | 76.6 |  |
| Source of control |  |  |  |  |  |  |  |  |  |  |  |  |  |  |  |  |  |
| HB | 24 | 1.33 (1.17-1.50) | <0.001 | 83.6 | 1.12 (0.99-1.27) | <0.001 | 76 | 1.34 (1.11-1.61) | <0.001 | 85.1 | 1.31 (1.17-1.47) | <0.001 | 78.6 | 1.17 (1.08-1.27) | <0.001 | 82.3 |  |
| PB | 14 | 1.22 (1.05-1.41) | <0.001 | 85.1 | 1.06 (0.86-1.31) | <0.001 | 86.1 | 1.24 (1.01-1.53) | <0.001 | 81 | 1.20 (1.04-1.39) | <0.001 | 81.3 | 1.10 (0.97-1.24) | <0.001 | 89.6 |  |
| Type of cancer |  |  |  |  |  |  |  |  |  |  |  |  |  |  |  |  |  |
| Gastric | 22 | 1.45 (1.27-1.66) | <0.001 | 82.5 | 1.14 (0.95-1.36) | <0.001 | 86.7 | 1.46 (1.17-1.83) | <0.001 | 86.8 | 1.43 (1.27-1.60) | <0.001 | 73.8 | 1.22 (1.10-1.35) | <0.001 | 87.6 |  |
| Bladder | 6 | 1.26 (1.19-1.32) | 0.414 | 0.2 | 1.18 (1.07-1.19) | 0.676 | 0 | 1.29 (1.21-1.38) | 0.001 | 0 | 1.25 (1.18-1.32) | 0.356 | 9.4 | 1.14 (1.11-1.18) | 0.423 | 0 |  |
| Breast | 2 | 1.03 (0.85-1.24) | 0.313 | 1.8 | 1.33 (0.95-1.85) | 0.191 | 41.4 | 1.29 (0.82-2.03) | 0.114 | 60 | 0.96 (0.79-1.16) | 0.374 | 0 | 1.09 (0.96-1.24) | 0.312 | 2.2 |  |
| Cervical | 1 | 0.85 (0.72-1.00) | - | - | 0.55 (0.39-0.79) | - | - | 0.53 (0.37-0.76) | - | - | 0.90 (0.76-1.07) | - | - | 0.83 (0.73-0.94) | - | - |  |
| Colorectal | 2 | 0.92 (0.64-1.33) | 0.233 | 29.6 | 1.22 (0.87-1.70) | 0.584 | 0 | 1.06 (0.72-1.56) | 0.788 | 0 | 0.86 (0.51-1.45) | 0.11 | 58.9 | 1.03 (0.84-1.26) | 0.704 | 0 |  |
| Esophageal | 2 | 0.67 (0.40-1.14) | 0.023 | 80.8 | 0.91 (0.73-1.13) | 0.919 | 0 | 0.75 (0.51-1.11) | 0.18 | 44.4 | 0.65 (0.36-1.18) | 0.013 | 82.1 | 0.83 (0.68-1.01) | 0.165 | 48.2 |  |
| Gallbladder | 2 | 1.22 (0.81-1.84) | 0.266 | 19.2 | 0.82 (0.50-1.35) | 0.217 | 34.4 | 0.96 (0.49-1.90) | 0.18 | 44.2 | 1.34 (0.96-1.88) | 0.513 | 0 | 0.96 (0.63-1.45) | 0.104 | 62.2 |  |
| Prostate | 1 | 1.27 (0.79-2.04) | - | - | 1.21 (0.74-1.98) | - | - | 1.38 (0.77-2.50) | - | - | 1.22 (0.74-2.01) | - | - | 1.17 (0.88-1.57) | - | - |  |
| ***rs2976392 G>A*** |  | (AG + AA) vs. GG | | | AA vs. (AG + GG) | | | AA vs. GG | | | AG vs. GG | | | A vs. G | | |  |
| All | 18 | 1.30 (1.11-1.53) | <0.001 | 84.2 | 1.12 (0.94-1.33) | <0.001 | 73.3 | 1.30 (0.99-1.70) | <0.001 | 85.2 | 1.28 (1.11-1.49) | <0.001 | 78.7 | 1.17 (1.05-1.31) | <0.001 | 83.1 |  |
| Ethnicity |  |  |  |  |  |  |  |  |  |  |  |  |  |  |  |  |  |
| Asian | 16 | 1.29 (1.09-1.53) | <0.001 | 84.7 | 1.06 (0.89-1.27) | <0.001 | 71.6 | 1.24 (0.93-1.64) | <0.001 | 85.3 | 1.29 (1.11-1.51) | <0.001 | 79.7 | 1.15 (1.03-1.29) | <0.001 | 82.7 |  |
| Caucasian | 2 | 1.43 (0.57-3.59 | 0.002 | 89.2 | 1.66 (0.88-3.13) | 0.024 | 80.3 | 1.90 (0.60-6.02) | 0.001 | 90.4 | 1.20 (0.56-2.54) | 0.02 | 81.6 | 1.39 (0.77-2.51) | 0.001 | 90.9 |  |
| Source of control |  |  |  |  |  |  |  |  |  |  |  |  |  |  |  |  |  |
| HB | 15 | 1.31 (1.06-1.60) | <0.001 | 86.6 | 1.14 (0.93-1.39) | <0.001 | 77.2 | 1.32 (0.96-1.83) | <0.001 | 87.5 | 1.28 (1.06-1.54) | <0.001 | 81.8 | 1.17 (1.03-1.34) | <0.001 | 85.7 |  |
| PB | 3 | 1.25 (1.09-1.42) | 0.314 | 13.6 | 1.03 (0.84-1.28) | 0.988 | 0 | 1.15 (0.92-1.43) | 0.889 | 0 | 1.26 (1.10-1.45) | 0.297 | 17.6 | 1.15 (1.05-1.25) | 0.426 | 0 |  |
| Type of cancer |  |  |  |  |  |  |  |  |  |  |  |  |  |  |  |  |  |
| Gastric | 13 | 1.43 (1.18-1.74) | <0.001 | 86.8 | 1.14 (0.91-1.43) | <0.001 | 79.8 | 1.41 (1.23-1.61) | <0.001 | 88.8 | 1.41 (1.19-1.67) | <0.001 | 80.1 | 1.24 (1.08-1.41) | <0.001 | 86.3 |  |
| Breast | 2 | 0.96 (0.80-1.16) | 0.662 | 0 | 1.15 (0.90-1.48) | 0.861 | 0 | 1.41 (1.23-1.61) | 0.666 | 0 | 0.93 (0.77-1.12) | 0.574 | 0 | 1.02 (0.90-1.16) | 0.923 | 0 |  |
| Colorectal | 1 | 0.90 (0.61-1.33) | - | - | 1.20 (0.80-1.79) | - | - | 1.41 (1.23-1.61) | - | - | 0.83 (0.54-1.25) | - | - | 1.02 (0.80-1.31) | - | - |  |
| Gallbladder | 1 | 0.78 (0.34-1.80) | - | - | 0.58 (0.28-1.20) | - | - | 1.41 (1.23-1.61) | - | - | 0.98 (0.40-2.35) | - | - | 0.72 (0.45-1.16) | - | - |  |
| Prostate | 1 | 1.25 (0.78-2.01) | - | - | 1.20 (0.73-1.95) | - | - | 1.41 (1.23-1.61) | - | - | 1.20 (0.73-1.99) | - | - | 1.16 (0.87-1.55) | - | - |  |
